# Examining domains of community health nurse satisfaction and motivation: results from a mixed-methods baseline evaluation in rural Ghana

**DOI:** 10.1186/s12960-015-0082-7

**Published:** 2015-10-08

**Authors:** Emma Sacks, Soumya Alva, Sophia Magalona, Linda Vesel

**Affiliations:** Department of International Health, Johns Hopkins School of Public Health, 615 N. Wolfe St., E8011, Baltimore, MD 21205 USA; USAID Maternal and Child Survival Program (MCSP)/ICF International, Washington, DC USA; John Snow, Inc., Rosslyn, VA USA; Concern Worldwide US, New York, NY USA

**Keywords:** Human resources for health, Community health nurses, Health worker motivation, Knowledge assessment, MCH, Maternal health, Neonatal health, Rural health workers, Mixed methods, Ghana

## Abstract

**Background:**

A strong health system requires a competent and caring workforce. A more satisfied and motivated health workforce should be more willing to serve in difficult areas, have lower turnover, and theoretically provide better care to patients. This paper examines the motivation, satisfaction, and correlation with clinical knowledge, of community health nurses (CHNs), a cadre of provider focused on maternal, newborn and child health in rural Ghana.

**Methods:**

This study employed three methods of evaluation. Two quantitative measurements were used: (1) a survey of health worker satisfaction and motivation and (2) a clinical knowledge assessment focusing on maternal, newborn and child health. Both were administered to all rostered CHNs working in the five sampled districts in the Greater Accra and Volta regions in Eastern Ghana (*N* = 205). Qualitative interviews (*N* = 29) and focus group discussions (*N* = 4) were held with selected CHNs in the same districts. These data were analysed using NVivo (Version 10) and Stata (Version 13.0) based on domains of extrinsic and intrinsic motivation including general satisfaction, work environment and access to resources, respect and recognition received and opportunities for advancement.

**Results:**

CHNs desired more training, especially those who were posted at the community level (a Community-based Health Planning and Services post or “CHPS”) versus at a health facility. CHNs working at CHPS believed their work to be more difficult than those posted at health facilities, due to challenges associated with foot travel to visit patients at home, and they were more likely to report having insufficient resources to do their jobs (48% vs 36%). However, CHNs posted at health facilities were more likely to report insufficient opportunities for career advancement than the CHPS nurses (49% vs 33%). CHNs generally reported good relationships with colleagues and being respected by patients but desired more respect from supervisors. The median score on the knowledge assessment was 78%. On average, subgroups of CHNs with different reported levels of satisfaction did not perform differently on the knowledge assessment.

**Conclusions:**

CHNs in Ghana were satisfied overall but desired more training, more guidance and supervision, fair pay and opportunities to advance in their career. Improving health worker satisfaction and morale may be important for health worker retention and certain aspects of care but may not have a significant influence on clinical knowledge or performance.

## Résumé en français

**Contexte:**

Un système de santé solide nécessite un personnel de santé compétent et attentionné.  Un personnel de santé plus satisfait et motivé sera mieux disposé à servir dans les zones difficiles, aura un meilleur taux de rétention, et théoriquement offrira des soins de meilleure qualité au patients.  Ce document examine la motivation, la satisfaction et leur corrélation avec les compétences cliniques des infirmières dénommées Community Health Nurses (CHNs), personnel en charge de la santé maternelle, néonatale et infantile dans le Ghana rural.

**Méthodologie:**

Cette étude utilise trois méthodes d'évaluation. Deux mesures quantitatives ont été utilisées: 1) une enquête de satisfaction et de motivation du personnel de santé, et 2) une évaluation des compétences cliniques sur la santé maternelle, néonatale et infantile, toutes deux appliquées à chaque CHN des cinq districts des régions Grand Accra et Volta dans l’est du Ghana (N=205). Des entretiens qualitatifs (N = 29) et discussions de groupe (N = 4) ont été organisés avec certaines CHNs dans ces mêmes districts. Ces données ont été analysées à l'aide de NVivo (version 10) et STATA (version 13), en se basant sur les domaines de motivation extrinsèque et intrinsèque comme la satisfaction générale, l’environnement de travail et l’accès aux ressources, le respect et la reconnaissance reçus, et les possibilités d'avancement.

**Résultats:**

Les CHNs ont exprimé un désir de formation, en particulier celles qui travaillent dans les communautés (CHPS) plutôt qu’au sein d’un centre de santé. Les CHNs travaillant dans les CHPS estiment que leur travail est plus pénible que celui des CHNs travaillant dans les centres de santé, ceci en raison des difficultés de déplacement effectué à pied pour les visites à domicile de patients, et dénoncent plus fréquemment l’insuffisance de ressources pour effectuer leur travail (48% vs 36%).  Cependant, les CHNs travaillant dans les centres de santé sont plus susceptibles que les CHNs de CHPS de déclarer insuffisantes les possibilités d’avancement professionnel (49% vs 33%).

Les CHNs font généralement état de bonnes relations avec les collègues et déclarent être respectées par les clients, mais demandent plus de respect de la part de leurs superviseurs.La note médiane en ce qui concerne l’évaluation des compétences était de 78%.  En moyenne, les sous-groupes avec niveaux différents de satisfaction ne se comportent pas différemment en ce qui concerne l’évaluation des compétences.

**Conclusion:**

Les CHNs au Ghana ont été satisfaits en général, mais ils ont désiré plus de formation, plus de directives et de la supervision, la salarie équitable et la possibilité d’avancer dans leurs carrières. Améliorer la satisfaction et le moral des travailleurs de la santé peut être important pour la rétention des travailleurs de la santé et pour des certains aspects des soins, mais ne peut pas avoir une influence sur le niveau de soins cliniques donnés.

## Background

According to the World Health Organization, the maternal mortality ratio in Ghana declined from 760 per 100 000 live births in 1990 to 380 per 100 000 live births in 2013 [1], and the under-five mortality ratio declined from 128 per 1000 live births to 78 per 1000 live births during the same time period [2]. Despite now being indexed as a middle-income country, Ghana’s maternal and under-five mortality ratios are still the same or higher than the average for low-income countries, which has been calculated as 230 per 100 000 live births and 76 per 1000 live births, respectively [3]. While Ghana has made admirable progress in maternal, newborn and child health to reach Millennium Development Goals 4 and 5, progress has been uneven throughout the country. A strong and competent health workforce is essential, not only in larger cities but also in isolated rural areas. Ghana’s current physician to population ratio is around 1:10 000, but physicians are concentrated at national level facilities with fewer than 15% working in district or sub-district levels, leaving the burden of care to nurses and community health agents [4].

Ghana has made efforts to implement many policies and strategies in the development of its human resources for health strategy through the provision of care at the community level. Ghana first implemented the Community-based Health Planning and Services (CHPS) programme as part of an experimental study by the Navrongo Health Research Centre in the 1990s. It rapidly grew into a national community health care programme aiming to improve the accessibility and quality of health and family planning care [5]. Supported by community leadership, CHPS compounds are Ghana’s primary strategy to extend health care provision to those who have been beyond the reach of the existing formal system. Community health nurses (CHNs), paid frontline health workers, are either posted at CHPS compounds or health facilities and provide community-based preventive and curative maternal, newborn and child health care while residing in the community [6].

The main role of the CHN is to focus on activities related to preventive health. The CHN assists public health nurses to work in the community to promote and maintain health especially among pregnant women, new mothers, infants and young children. CHNs conduct home visits and outreach activities in the community and provide health education. CHNs obtain a Certificate in Community Health Nursing as part of pre-service training; this certificate is obtained after completing a 2-year curriculum post secondary school. After 3 to 5 years of service, CHNs are able to enrol for higher education that enables them to become a midwife or public health nurse (PHN). Enrolled and registered nurses have higher qualifications than CHNs that enable them to do more advanced clinical work in health facilities.

CHNs are supervised by persons in charge at the CHPS compound or by sub-district heads or public health nurses at the district level. CHNs are considered “close to community” providers and live in the patient catchment areas, which may be different from their home districts. These more rural areas may also be disconnected from larger health centres, professional networks and opportunities for advancement. It has been theorized that improved supervision and better relationships among the CHNs may have an impact on job satisfaction and motivation [7, 8], as well as clinical knowledge and performance [9]. Some studies have found that collaborative skill-building can improve both performance and inter-personal relationships among health workers [10, 11]. These questions are especially relevant to Ghanaian CHNs, given that these health workers live in environments of professional isolation and limited support.

In 2002, the Ministry of Health developed a 5-year human resource plan to guide implementing agencies, both public and private [12]. It focused on increasing production and retention of staff and equipping them with the relevant tools to provide health care. However, the outlined strategies in the document were not fully implemented, resulting in varied successes that led to existing human resource gaps in the health sector. The human resources for health strategy calls for CHNs to receive regular supportive visits from supervisors. One hypothesized pathway to improving performance and retention of health workers is through more encouragement, with the assumption that a more satisfied and motivated workforce will provide better clinical care, encourage more health care utilization by patients and stay in their jobs longer [12]. However, not all CHNs receive adequate or regular supervision, and it is not clear that these inputs are sufficient [4, 9]. Understanding factors that influence health workers’ satisfaction and motivation is relevant in this context for understanding their retention and performance [13]. Health worker motivation is driven by individual determinants such as workers’ personal and professional goals as well as the relationship between the worker and the work environment and broader organizational and societal factors [14, 15]. The role of these factors is complex, affecting both aspects of intrinsic and extrinsic motivation among individuals, and varies widely by local context [16, 17].

This paper presents findings from an assessment of health worker motivation and clinical knowledge among CHNs in five rural districts in Ghana, focusing on various domains of satisfaction and motivation, both extrinsic and intrinsic. This study was conducted as part of a baseline assessment in advance of the implementation of the Care Community Hub (CCH) project by Concern Worldwide US’s *Innovations for Maternal, Newborn and Child Health* initiative and The Grameen Foundation in Ghana. The aim of the project is to address barriers in health worker motivation through the use of a smartphone application that improves connectivity and communication between CHNs, increases their linkages and interactions with a professional network and supervisors and provides clinical refreshers, point of care tools and diagnostic aids, along with a calendar and daily motivational quotes.

## Methods

This study utilized a mixed methods approach with quantitative and qualitative data collection and analysis. Data collection took place in five districts – Ningo Prampram and Ada East and Ada West in the Great Accra region and South Dayi and South Tongu in the Volta region. For the purpose of this assessment, data from Ada East and Ada West were combined, as they had been one administrative district that was recently divided.

The quantitative data collection included two instruments. One was a survey on job satisfaction, motivation, relationships, communication with peers and supervisors, career goals and challenges faced by CHNs, which was developed based on formative research conducted in the regions. The second tool was a clinical knowledge assessment, covering the areas of antenatal care, newborn care, infant feeding, immunization, human immunodeficiency virus (HIV) and family planning, with questions based on the national training curriculum for CHNs. The job satisfaction survey and clinical knowledge assessment were administered to all CHNs (*N* = 205) in the selected districts between June and August 2014 prior to the CHNs receiving mobile phones or any training by the implementing organizations for the CCH project.

The qualitative data collection included in-depth interviews (N=29) and focus group discussions (N=4; 23 individual participants) with CHNs to better understand their professional roles, work burdens and challenges, job satisfaction and relationships with peers and supervisors. Qualitative data collection was conducted in the selected districts in May and June 2014. CHNs were selected based on established criteria to represent the range of sexes, seniority, years of experience and posting in a CHPS compound or health facility. Supervisors were also interviewed, but data from supervisor interviews were not included in the analysis presented in this paper.

All respondents were asked to complete an informed consent form before taking either of the self-administered paper surveys. In all cases, the satisfaction survey was administered prior to the knowledge assessment. ID numbers linking the two surveys were assigned so that data from the two surveys could be interlinked; however, names or other identifiers of the participants were not collected.

This study was approved by the Institutional Review Board (IRB) at John Snow Inc. and the Ethical Review Committee at the Ghana Health Service (GHS-ERC: 07/09/13).

### Data analysis

Quantitative data from the motivation and satisfaction survey and knowledge assessment were entered into Excel and transferred to Stata (version 13) for analysis, and descriptive statistics were generated. Statistical comparisons were done using the chi-square analysis or Fischer’s exact test for the satisfaction and motivation analyses and the Kruskal–Wallis non-parametric test for the knowledge assessment scores. Results of significance at the 95% confidence level are presented in the tables. Data are reported only for nurses who responded to both the assessments. Findings from the qualitative data collection were compiled in the form of interview transcripts and summary notes. All data were coded in NVivo (Version 10), and findings were extracted based on predefined codes, using thematic content analysis techniques [18].

We present descriptive results of satisfaction and motivation among CHNs in the sampled districts in Ghana, measured across various domains of extrinsic and intrinsic motivation. While characteristics such as job desirability and compensation, work environment and access to resources are the extrinsic motivation needs of CHNs, respect from supervisors, patients and peers and opportunities for career advancement would be expected to provide them intrinsic motivation to perform their jobs better. These findings are presented by district and by posting type: either CHPS compound or health facility.

In most of the quantitative assessments, respondents were asked to provide the “most important” choice out of a list; thus, our analysis of the most common responses does not preclude other responses also being relevant. The qualitative data explore CHN perceptions more broadly.

### Findings

#### Demographics

As shown in Table 1, the CHNs interviewed were relatively homogenous, with approximately 90% female, well over half younger than 30 years old and less than half married in every district. There were very few significant differences in responses due to sex, marital status or age, but important variations are presented here. As this study was restricted to CHNs, none of the participants held higher level nursing or midwifery degrees.

**Table 1:** **Community health nurse (CHN) demographics (%)**

	**Greater Accra region**		**Volta region**		**Total**
	**Ada East/West**	**Ningo Prampram**	**South Tongu**	**South Dayi**	
*N*	54	44	46	42	186
Sex					
Female	94.4	90.7	89.1	92.7	91.8
Male	5.6	9.3	10.9	7.3	8.2
Age					
18–24 years	3.7	9.3	19.6	9.5	10.3
25–30 years	75.9	65.1	65.2	64.3	68.1
31–39 years	16.7	9.3	15.2	19.0	15.1
40–49 years	1.9	2.3	0.0	7.1	2.7
50 years or older	1.9	14.0	0.0	0.0	3.8
Marital status					
Married	33.3	47.7	37.0	34.1	37.8
Single	63.0	50.0	63.0	58.5	58.9
Divorced	0.0	2.3	0.0	0.0	0.5
Living with someone	1.9	0.0	0.0	7.3	2.2
Widowed	1.9	0.0	0.0	0.0	0.5
Health facility					
CHPS	44.4	77.3	60.9	23.8	51.6
Health centre	55.6	22.7	39.1	76.2	48.4
Are you the CHN in-charge?					
Yes	29.6	9.1	23.3	21.4	21.3
No	70.4	90.9	76.7	78.6	78.7
Hometown in the same district					
Yes	7.4	2.3	13.0	4.8	7.0
No	92.6	97.7	87.0	95.2	93.0
Currently live with any family member					
Yes	40.7	48.8	34.8	47.6	42.7
No	59.3	51.2	65.2	52.4	57.3

The distribution between placements at CHPS compounds versus at health facilities varied between the surveyed districts. In Ningo Prampram and South Tongu, more than half of the CHNs were placed at CHPS compounds, while in Ada East and West, the split was closer to half, and in South Dayi (the furthest district from Accra), over 75% of the CHNs were placed in CHPS compounds. In every district, CHNs who were themselves the person in charge at their health facility/CHPS made up less than 30% of our sample. Almost all of the CHNs were posted to different districts than their home districts and less than half had a family member living with them.

#### CHNs overall satisfaction with their jobs

More than half of participants (56%) responded that they were either “satisfied” or “very satisfied” with their jobs; analysis of satisfaction stratified by the demographic categories in Table 1 did not reveal any significant differences (Table 2). By district, satisfaction levels were higher in Ada East and West compared to the other districts. Differences in satisfaction levels between those placed at CHPS compounds versus at health facilities were small.

**Table 2:** **CHN level of job satisfaction (%)**

	**Greater Accra region**		**Volta region**		**Health facility**		**Total**
	**Ada East/West**	**Ningo Prampram**	**South Tongu**	**South Dayi**	**CHPS compound**	**Health centre**	
Overall, how satisfied are you with your job?							
*N*	*54*	*44*	*41*	*45*	*95*	*89*	*184*
Very satisfied	11.1	9.1	8.9	12.2	9.5	11.2	10.3
Satisfied	51.9	40.9	44.4	46.3	48.4	43.8	46.2
Somewhat satisfied	25.9	34.1	33.3	26.8	29.5	30.3	29.9
Not satisfied	11.1	15.9	13.3	14.6	12.6	14.6	13.6
Being a CHN is a desirable job							
Agree	87.0	92.8	89.1	85.7	92.6	84.4	88.6
Neutral	11.1	4.8	4.3	9.5	4.3	11.1	7.6
Disagree	1.9	2.4	6.5	4.8	3.2	4.4	3.8
I am paid fairly for the work that I do							
Agree	16.7	25.6	20.0	7.5	22.0	12.6	17.4
Neutral	18.5	23.1	17.8	20	16.5	23.0	19.7
Disagree	64.8	51.2	62.2	72.5	61.6	64.4	63.0
I want to continue working at this health facility for quite some time							
Agree	51.9	76.9	56.5	60.0	60.5	60.2	60.3
Neutral	24.1	5.1	28.3	30.0	19.8	25.0	22.3
Disagree	24.1	18.0	15.2	10.0	19.8	14.8	17.3

CHNs largely agreed that, for someone with their skill set, they had a desirable job. Despite being generally satisfied, they identified specific areas of dissatisfaction. More than 60% of CHNs indicated that they were not satisfied with the pay and did not believe that they were being compensated fairly. In South Dayi, in particular, almost 73% stated that their compensation was not fair. Half of CHNs over age 50 agreed strongly that they were paid fairly, compared to less than 15% of CHNs under age 50. Married CHNs were more likely to rank insufficient resources to do their jobs as their primary difficulties, compared to unmarried CHNs, who were more likely to rank insufficient opportunities for career advancement. This pattern was similar for older and younger CHNs, with younger respondents ranking insufficient career advancement opportunities highest and older CHNs ranking insufficient resources as the most problematic. Although the sample size of unmarried CHNs living with a long-term partner was very small, compared with both married and single counterparts, this group ranked “poor living conditions” more highly.

Sixty per cent of the CHNs surveyed indicated that they wanted to continue working at their current health post, with the highest level of agreement from CHNs in Ningo Prampram (77%), as compared to other districts. There was no difference observed between CHNs from CHPS compounds and CHNs from health facilities.

#### Suitable work environment

The survey asked respondents to rank perceived barriers that affected their ability to perform their jobs. In all districts, more than 80% of CHNs rated lack of resources (such as transport, commodities and phones) and limited opportunities for career advancement as the two most important barriers to performing their jobs well (Table 3). In South Dayi, in particular, more than half of the nurses felt that lack of opportunities for advancement was their most important concern. CHNs working at CHPS compounds were more likely to report insufficient resources to do their jobs as the primary barrier than those posted at health facilities (48% vs 36%); CHNs posted at health facilities were more likely to report insufficient opportunities for career advancement as their primary barrier than the CHPS nurses (49% vs 33%). In comparison, in no district did social isolation emerge as the most important barrier.

**Table 3:** **CHN-perceived job barriers (%)**

	**Greater Accra region**		**Volta region**		**Health facility**		**Total**
	**Ada East/West**	**Ningo Prampram**	**South Tongu**	**South Dayi**	**CHPS compound**	**Health centre**	
*N*	52	40	45	42	91	88	179
What I struggle with the most in my job as a CHN is:							
Inadequate pay	3.8	5.0	6.7	4.8	4.4	5.7	5.0
Inadequate support/guidance from my supervisors	0.0	5.0	4.4	2.4	4.4	1.1	2.8
Poor living conditions	5.8	2.5	2.2	2.4	5.5	1.1	3.4
Inadequate opportunities to advance my career	40.4	40.0	31.1	52.4	33.0	48.9	40.8
Inadequate resources to do my job (transport, commodities, phones, for example)	46.2	45.0	48.9	28.6	48.4	36.4	42.5
Poor mobile coverage/bad network to reach the people I need	1.9	0.0	4.4	4.8	2.2	3.4	2.8
Being isolated or having difficulty finding friends or partners	1.9	0.0	0.0	2.4	1.1	1.1	1.1
None of the above	0.0	2.5	2.2	2.4	1.1	2.3	1.7

Qualitative data from both in-depth interviews (IDIs) and focus group discussions (FGDs) supported these findings. When asked about their work environment, very few CHNs talked about positive aspects of their job but rather expressed frustration. CHNs complained about the lack of adequate equipment - particularly the lack of functional refrigerators for vaccines and weighing scales for infants - as a challenge that prevented them from providing necessary services to their patients. A few CHNs also mentioned that staff shortages and insufficient space to do their work were important issues.

“We don’t have some of the equipment; you requested but [it] is not available and that makes the work difficult and [so you are] not able to render service to the clients.” (CHN from Ada West, FGD)

“The department lacks some clinical equipment and the few which are there such as the toddlers’ scale is not working, it gives wrong or inconsistent measurement. Also, the department has only one sphygmomanometer [apparatus to measure blood pressure] which is used by all nurses.” (CHN from Ningo Prampram, IDI)

“Without the basic equipment that we mentioned, doing most of our routines becomes difficult.” (CHN from South Dayi, FGD)

Some other challenges at work that nurses mentioned include transportation issues, having to use personal money to pay for transport, seasonal challenges with rain or floods, staff shortages, lack of clinical refreshers and language issues with patients. A few nurses also complained about the lack of housing for themselves, which the CHPS programme was expected to provide.

#### Respect and recognition

Table 4 presents data on CHNs’ perceptions of the respect and recognition they receive from the patients and others they interact with as part of their job. In general, the CHNs reported that their interactions with their communities were very rewarding. More than 65% of the CHNs felt that the respect of patients was the most important type of respect that they desired, and the respect of other community members, including the families of patients, was second. Overall, about three quarters of CHNs stated that they felt respected by their patients. More of the CHNs posted at CHPS compounds reported feeling respected by their patients than CHNs posted at health facilities, but the difference was not significant. All of the CHNs over 50 years old agreed that they felt respected by their patients, while a small percentage of younger CHNs were neutral or disagreed.

**Table 4:** **Perceived respect by CHNs in their job (%)**

	**Greater Accra region**		**Volta region**		**Health facility**		**Total**
	**Ada East/West**	**Ningo Prampram**	**South Tongu**	**South Dayi**	**CHPS compound**	**Health centre**	
*N*	54	42	45	41	95	87	182
Whose respect is most important to you?							
Clients/patients	64.8	61.0	55.6	80.5	62.8	67.8	65.2
Supervisors	13.0	9.8	8.9	2.4	8.5	9.2	8.8
Other CHNs	1.9	0.0	0.0	2.4	1.1	1.1	1.1
Community members	18.5	29.3	33.3	14.6	27.7	19.5	23.8
Nobody	1.9	0.0	2.2	0.0	0	2.3	1.1
I feel respected by clients							
Agree	73.2	86.3	89.1	78.1	84.4	78.4	75.6
Neutral	20.8	13.6	10.9	22.0	14.6	19.3	16.9
Disagree	5.7	0.0	0.0	0.0	1	2.3	1.6

Despite the high volume of their interaction with community members as part of their jobs, the CHNs generally did not view their patients as a burden. Many CHNs felt that community members respected them and were appreciative of their services and were glad that patients often confide in them with their problems.

“I feel respected and loved because sometime when I walk in the community and the community members call me: “Ma’am nurse, how are you? How are you faring?” (CHN from Ningo Prampram, FGD)

“It’s because of them [that] we are in the community… and for you being a nurse they will by all means come to you if they are finding any problem or difficulties.” (CHN from South Dayi, IDI)

When asked what they enjoyed most about their interactions with patients, more than two thirds of the nurses stated that they most enjoyed listening to their patients’ problems and finding workable solutions (Table 5). The other three aspects CHNs enjoyed when they interacted with patients were feeling proud that they were able to serve the community, figuring out medical issues and knowing how to treat patients. These aspects were reported to the same degree across all of the districts. However, women were slightly more likely than men to report being motivated by being able to help (“listening to patients’ problems and finding workable solutions”), and men were slightly more likely than women to report wanting to solve medical issues and feel respected by their patients.

**Table 5:** **Positive and negative aspects of client interaction perceived by CHNs (%)**

	**Greater Accra**		**Volta**		**Health facility**		**Total**
	**Ada East/West**	**Ningo Prampram**	**South Tongu**	**South Dayi**	**CHPS compound**	**Health centre**	
*N*	54	44	45	42	95	90	185
What I enjoy most from my interactions with clients is…							
Feeling proud that I am able to serve the community	13.2	13.6	8.9	9.5	10.5	12.4	11.4
Listening to their problems, and finding workable solutions for them	73.6	75.0	73.3	88.1	73.7	80.9	77.2
Figuring out what their medical issues are, and knowing how to treat them	13.2	9.1	11.1	2.4	11.6	6.7	9.2
Feeling respected or admired by clients	0.0	2.3	6.7	0.0	4.2	0	2.2
What I find most difficult from my interactions with clients is…							
When they argue or disagree with my recommendations	13.5	16.3	23.9	4.8	18.1	11.2	14.8
I feel I don’t have the right resources to help them	65.4	74.4	58.7	88.1	67	75.3	71.0
I don’t have the knowledge on what to do	3.8	4.7	6.5	2.4	5.3	3.4	4.4
I have no challenges in working with the community	17.3	4.7	10.9	4.8	9.6	10.1	9.8

Although they describe their relationship as generally positive, most CHNs did mention some difficulties in working with their patients. CHNs complained that they did not have the right resources to work with their patients; this was consistent across all the districts. Close to a quarter of the nurses in South Tongu also stated that they found it difficult when patients argued or disagreed with their recommendations. Some challenges reported by nurses included language barriers, patients’ lack of understanding and time management when they interacted with patients.

“I can’t speak the language here very well. The dialect the people are speaking here, I can’t express myself. That’s another aspect because when you are living with people, you have to understand their language… [so when they] come to you [you can] give them good counseling.” (CHN from Ada, IDI)

CHNs reported being frustrated by patients that either did not understand the information being provided to them or chose to disregard the nurses’ advice after they had taken the time to sit with them.

“Some of them, the education we give them, they don’t practice it, so they come with the same problem over and over again.” (CHN from South Dayi, IDI)

Some patients also did not show up when nurses had scheduled to meet them, resulting in the nurses wasting time during the day.

“[Y]ou tell them to come to antenatal on Wednesday, they will come on Monday. Somebody she is not sick but she will come on Monday. You know, it makes the work difficult for you, because you wouldn’t be around; you’ve gone for outreach and you’ve come, you understand? And she will say, “I came there and you guys were not around.” (CHN from Ada East, IDI)

CHNs reported that some community members did not view the position of CHNs as well as other health workers, which made it difficult for them to do their work.

“We as a community health nurse working in the community, the people over there, they regard the general [enrolled or registered] nurses [better] than us. They think we don’t know so they discriminate about us a lot. Even some houses when you enter … even when you greet them, they are not ready to respond.” (CHN from South Dayi, FGD)

In addition, CHNs also noted that they were often expected by the community to perform a wider range of clinical procedures than they were trained to do and felt there was a misunderstanding about their abilities and role.

#### Opportunities and resources for advancement

As shown in Table 6, less than 10% of CHNs in every district imagined themselves as CHNs 5 years in the future. Sixty to 90% of the CHNs stated that they wanted to become midwives or public health nurses in 5 years, requiring education and promotion. A slightly higher percentage of nurses posted at CHPS compounds desired more advanced work in 5 years than those posted to health facilities.

**Table 6:** **CHNs’ perception of their future work opportunities (%)**

	**Greater Accra region**		**Volta region**		**Health facility**		**Total**
	**Ada East/West**	**Ningo Prampram**	**South Tongu**	**South Dayi**	**CHPS compound**	**Health centre**	
*N*	54	39	46	40	91	88	179
In 5 years, I see myself as a…*							
CHN/CHO	0.0	7.1	8.7	7.5	4.9	6.3	5.6
Midwife	29.2	32.1	15.2	20.0	23.2	23.8	23.5
Public health nurse	62.5	60.7	47.8	62.5	62.2	53.8	58.0
Medical Assistant	6.3	0.0	17.4	5.0	7.3	8.8	8.0
Other position in public health	2.1	0.0	10.9	5.0	2.4	7.5	4.9
I have easy access to resources such as continuing education and professional meetings that will help build my skills for this job							
Agree	42.3	31.6	36.3	45.0	37.8	40.5	39.1
Neutral	13.5	15.8	11.4	7.5	12.2	11.9	12.1
Disagree	44.2	52.6	52.3	47.5	50	47.6	48.9
To be happy working as a CHN, I most need/want:							
More training	62.3	73.7	75.6	76.9	72.7	70.1	71.4
More money	9.4	2.6	2.2	7.7	4.5	6.9	5.7
Better supervision	3.8	7.9	15.6	5.1	8.0	8.1	8.0
Respect from supervisors	22.6	13.2	6.7	10.3	12.5	14.9	13.7
Respect from other CHNs	1.9	0.0	0.0	0.0	1.1	0.0	0.6
Nothing. I have everything.	0.0	2.6	0.0	0.0	1.1	0.0	0.6
In order to achieve my future goals, the most important thing I would need is:*							
In-service training opportunities	42.3	63.9	26.7	66.7	46.6	50.0	48.3
Training materials (books or online) to strengthen my skillsets	21.2	22.2	6.7	12.8	17.0	14.3	15.7
A good working relationship with supervisors who can advise you in achieving my goals	34.6	13.9	66.7	20.5	36.4	34.5	35.5
I am not sure and I feel stuck in my situation	1.9	0.0	0.0	0.0	0.0	1.2	0.6

*p value <0.05 for district comparison.

CHNs’ views on whether they had enough resources for professional advancement were mixed. Only 39% agreed that they had adequate resources for their professional advancement; almost 50% disagreed with the statement. In order to achieve their future goals, their two most important needs were in-service training opportunities (48%) and a better relationship with their supervisors (35%). Particularly in South Tongu, more than 60% of nurses expressed the need for a better working relationship with their supervisors, whereas in Ningo Prampram and South Dayi, the need for in-service training was most strongly expressed (more than 60%). More women than men ranked the need for more training as most important to their professional development, while more men ranked the importance of respect from their supervisors. Nurses from both CHPS compounds and from health facilities demonstrated similar patterns of responses.

Receiving promotions to a higher level of medical professional was also stated as a challenge, given the current advancement system. CHNs reported that after 5 years, they could be eligible for further study (to become a public health nurse or midwife) and would leave their posts to do so. However, their current career trajectories do not include a pathway to become enrolled or registered nurses due to the prerequisites needed; thus, CHNs would have to leave nursing and obtain a higher level general diploma before beginning a new course of nursing studies.

“So they should have the degree, so that maybe if you want to further your education, you will still be a community health nurse, but, maybe you will have a degree or something. Because right now, if you want to further [your career]… you leave community health nursing, or maybe I’m a diploma holder, and I want to further, I can’t go and do the degree…I have to divert [to get a higher degree first].” (CHN from Ningo Prampram)

Older CHNs were also at a disadvantage in the current system, because many of them typically did not want to go back to school.

“Some of our colleagues are old in the system. They cannot go to any school or advance in any education again. So what I would like them, our in-charges, to do is to find some ways to upgrade them so that they can also be happy, because they are in the system for long.” (CHN from Ningo Prampram)

The lack of opportunities for advancement was frustrating to many nurses; they strongly desired the option of staying as a CHN and working towards an advanced degree.

#### Clinical knowledge assessment

The clinical knowledge assessment covered questions on antenatal care, newborn care, infant feeding, immunizations, HIV and family planning. The average score on the assessment was 78.2%, with almost no difference between district averages (range: 76.5% in South Dayi to 79.2% in Ningo Prampram). Highest scores were earned on questions related to immunizations and lowest scores on sections related to antenatal and newborn care.

There were no statistically significant differences by sex, age or marital status. CHNs posted at health facilities performed slightly better than their counterparts posted to CHPS compounds, but the difference was not significant.

As shown in Table 7, subgroups of respondents were categorized by their reported levels of satisfaction, as measured by the satisfaction and motivation survey previously administered and cross-tabulated against their scores on the clinical knowledge assessment. CHNs reporting higher satisfaction performed slightly better on the assessment, especially in the areas of HIV knowledge and newborn care; however, the difference was not significant.

**Table 7:** **Percent correct in each section of the clinical knowledge assessment by reported level of overall satisfaction**

	**Satisfaction**		**Posting location**		**Total**
	**Satisfied**	**Not satisfied**	**CHPS compound**	**Health facility**	
*N*	104	80	96	89	185
Antenatal care	71.3	69.6	69.9	71.4	70.7
Postnatal care	76.1	74.1	74.6	76.1	75.3
Immunizations	95.3	94.3	95.2	94.6	94.9
Infant care	74.7	72.0	73.5	73.8	73.6
HIV	77.2	72.8	71.6	79.3	75.3
Family planning	80.1	77.6	78.2	80.1	79.1
Total	79.1	66.7	77.2	79.2	78.2

## Discussion

Overall, health workers reported being fairly satisfied at their jobs, with their biggest challenges being lack of resources to do their jobs, lack of opportunities for advancement and the feeling that they were not compensated fairly for their work. CHNs were generally young; most were posted to districts different than their hometowns but did not rank social isolation as their primary challenges and more than half said they wanted to continue working in their current post. Social isolation was expected to be an important barrier [9], yet professional isolation, along with the lack of resources, consistently ranked higher. CHNs placed at CHPS compounds reported more dissatisfaction with the available resources to carry out their duties than their health facility counterparts, suggesting that more emphasis needs to be placed on equipping these CHNs, who provide much of the maternal, newborn and child health care outside of facilities, in community members’ homes.

CHNs reported generally positive interactions with patients and enjoyment in helping community members. More often than not, CHNs wanted better resources to help their patients and expressed few frustrations with the patients themselves. They did, however, report challenges with the language barrier in some cases, low health literacy of patients and inefficient appointment scheduling. CHNs reported feeling rewarded from their interactions with patients and suggested that they get more appreciation and gratitude from their patients than from their supervisors. The supervision structure in the Ghana Health Service is not standard across districts; further analysis is currently underway to understand the frequency and content of supervision for these CHNs, including different experiences based on sex, age and marital status.

While CHNs generally saw their job as desirable given their skill set, they believed their work to be more logistically and physically difficult than other health care workers because of the amount of time spent outside, traveling to patients’ houses. This sentiment was strongest from CHNs based at CHPS compounds, although those posted to health facilities reported spending much of their time doing outreach as well. Very few of the respondents planned to stay as CHNs for more than 5 years and expressed frustration at the difficulties in obtaining promotions to other types of health care work.

Overall, the CHPS model is likely more sustainable than a volunteer-based community model, as the budget for health workers to be compensated and supported is built into the national budget, rather than relying on variable short-term project funding [6]. In a Cochrane review of volunteer health workers, it was suggested that stronger connections with and support from the health system would lend credibility to community health workers [19]; the CHPS model demonstrates a successful incorporation of community-based nurses. Yet, the rapid national expansion of the CHPS programme may have contributed to some of the CHNs’ frustrations, as the time was not taken to train health workers from the target communities who would have spoken the same language, facilities were not upgraded prior to the increase of health workers and communities were not prepared to provide free housing to CHNs, as originally planned. While other cadres of health workers in Ghana have expressed high levels of attrition intention [20], CHNs might be comparing themselves to community health volunteers and thus are more satisfied with their compensation than other formal providers, who do not compare themselves to volunteers.

The provision of non-financial benefits should be considered as a strategy to improve retention as well. A study in India found that non-financial motivators, such as work environment and subsidized housing, were key to health worker satisfaction [13]. A study in Kenya identified work environment – the physical state of the health facility, as well as training, job security, supervisor support and a manageable workload – as important non-financial motivators [21]. In Ghanaian facilities, specifically, the facility managers’ overall attitude towards quality improvement and patient safety was correlated with higher health worker satisfaction [4]. Other possible motivators to be considered by policymakers might be work for a spouse or family member and free or subsidized schooling for health workers’ children in rural areas [22].

Clear and reasonable expectations for advancement are essential for maintaining good will and satisfaction. A study conducted in Kenyan hospitals identified that clear “expectations in terms of promotions, performance appraisal processes, and good communication” were key in creating an enabling work environment and a motivated workforce [23], and a study in Tanzanian primary care centres found that the staff desired “more structured and supportive supervision from managers, and improved transparency in career development opportunities” [24]. Almost all of our respondents desired a promotion within the succeeding 5 years, suggesting that opportunities and resources for advancement are vital for retaining talented staff. The only exception was older nurses who were planning to retire. As the current system for professional advancement in the Ghana Health Service is tracked between cadres, CHNs must take certain courses over again and spend significant time away from work for school in order to advance. While CHNs sometimes have the option of attending workshops, these sessions are infrequent and irregular, may not count as education credit and only a limited number of nurses are able to attend. Thus, the use of online modalities, short training courses or on-site certifications could be explored as potential paths to promotion for CHNs not wishing to start a new career path. Studies in other countries have identified the importance of providing opportunities for skill upgrades [13], although further research needs to be conducted on the lasting clinical effects of workshops and trainings, as well as the effect of clinical refreshers delivered online or by smartphone application.

The limitations of this study include the fact that satisfaction levels were all self-reported. There may have been desirability bias present, although participants were willing to openly state their dissatisfactions and talk about their challenges. All rostered CHNs who were available during the time period were surveyed, but long-term absentees, individuals on leave or staff away from their posts for training or educational purposes were excluded, which could have influenced the responses.

This paper does not address the complex psychology underlying the interplay of expectations, goals and satisfaction, but it bears mention that community health workers in the more deprived areas might expect fewer resources to be available and subsequently have different levels of satisfaction with what is offered. Different managers will have and express varying expectations of health workers and much of satisfaction could rely on individual personalities and desires. Additionally, as one deficiency is addressed, there will undoubtedly be other dissatisfactions [25, 26]. For example, that older CHNs were more likely to rank insufficient resources to do their jobs as a primary concern compared with younger CHNs does not indicate that younger CHNs believed they had sufficient resources, rather that they had other concerns, such as career advancement, which were not shared with the older CHNs who planned to retire in the near future. Our study was limited by a relatively homogenous sample, but more research is needed to investigate potential differences in satisfaction and motivation between sexes, different age groups and by other socio-demographic variables.

While it may ultimately be valuable to address the personal satisfactions of health workers to keep them happy, the implications for policy rest more on providing the tools and incentives necessary for these health workers to stay in their jobs and to perform them to a reasonable standard. Once individual health workers feel that they are paid an adequate amount, they may desire salary raises or bonuses, yet their job performance is more likely to depend on their access to clinical information and medical equipment. Health worker retention strategies should focus first on providing health workers (and facilities) with the necessary equipment and medications needed, including telephones and vehicles, and providing at least the minimum level of salary and incentive to retain workers in their posts.

More importantly, additional research is needed to understand the impact of satisfaction and motivation on clinical performance in delivering care for patients in a holistic sense. Our findings were consistent with a recent systematic review on motivation which found that in addition to fair pay, recognition and adequate resources are essential for retention [27]. But further analysis must be conducted on the various types of support and incentives that can improve satisfaction and motivation as they relate to performance. Our data showed no correlation between satisfaction level and clinical knowledge scores. Thus, improving satisfaction or motivation may not be sufficient to improve clinical knowledge and possibly, in turn, performance. Our data indicate the important prioritizations made by health workers with regard to their satisfaction and motivation. Yet, the links between knowledge, performance, satisfaction and retention must be studied explicitly, so as to design policies that can have the greatest effect on the health of the population. CHNs largely desired fair compensation and sufficient resources to carry out their work. Thus, system-wide improvements are necessary: equipping and supervising health workers must be done through support at the facility, district and national levels. A broader focus on strengthening the entire health system, including community components, is imperative for supporting and maintaining a satisfied and motivated community health workforce.

## Abbreviations

CCH: Care Community Hub
CHN: Community health nurse
CHPS: Community-based Health Planning and Services
ERC: Ethical Review Committee
FGD: Focus group discussion
GHS: Ghana Health Service
HIV: Human immunodeficiency virus
IDI: In-depth interview
IRB: Institutional Review Board
JSI: John Snow Inc.
